# Influence of Different Adhesives and Surface Treatments on Shear and Tensile Bond Strength and Microleakage with Micro-CT of Repaired Bulk-Fill Composites

**DOI:** 10.3390/polym17121680

**Published:** 2025-06-17

**Authors:** Handan Yıldırım-Işık, Mediha Büyükgöze-Dindar

**Affiliations:** 1 Department of Restorative Dentistry, Faculty of Dentistry, Istanbul Beykent University, 34500 Istanbul, Turkey; handanyildirim@beykent.edu.tr; 2 Heath Services Vocational School, Trakya University, 22030 Edirne, Turkey

**Keywords:** composite repair, dental bonding, dental leakage, shear strength, tensile strength

## Abstract

The repair of defective composite restorations, particularly bulk-fill composites, offers a conservative alternative to complete replacement. However, establishing durable adhesion between aged and fresh composites remains a clinical challenge due to the altered surface properties of aged materials. This in vitro study investigated the effects of different surface treatment protocols (no treatment, diamond bur roughening, and air abrasion) and adhesive systems (G-Premio Bond, Clearfil SE Bond, and Adper Single Bond 2) on the shear bond strength (µSBS), tensile bond strength (µTBS), and microleakage of repaired bulk-fill composites. Results demonstrated that both surface treatment and adhesive type significantly affected bond strength (*p* < 0.05). Mechanical surface treatments, particularly diamond bur roughening and air abrasion, enhanced µSBS and µTBS compared to untreated controls. The highest µSBS and µTBS values were observed with diamond bur treatment combined with Adper Single Bond 2, reaching mean values of 25.8 ± 2.1 MPa and 28.3 ± 1.8 MPa, respectively. Air abrasion with Clearfil SE Bond also significantly increased bond strengths (µSBS: 22.1 ± 2.0 MPa; µTBS: 23.5 ± 1.7 MPa) relative to no treatment (*p* < 0.05). Micro-computed tomography analysis revealed that Clearfil SE Bond following diamond bur roughening resulted in the lowest microleakage scores, with a mean leakage volume of 0.12 ± 0.04 µm. These findings underscore the importance of mechanical surface conditioning and appropriate adhesive selection to enhance both bond strength and sealing efficacy in composite repair procedures.

## 1. Introduction

Composite resin restorations, akin to all restorative materials, exhibit limited clinical longevity, with an annual failure rate of approximately 2.2% [1]. Mechanical failures (e.g., fractures) and biological complications (notably secondary caries) are primary causes of failure, while factors such as occlusal wear, marginal degradation, and discoloration may further compromise long-term performance [2,3].

Compellingly, clinical evidence indicates that in many cases, a significant portion of the restoration—often exceeding 80%—remains intact, with deterioration limited to localized areas [2]. Consequently, complete replacement may be regarded as unnecessarily invasive, contributing to a restorative cycle—often referred to as the ‘restoration death spiral’—that progressively weakens the tooth structure [3,4] and increases the risk of irreversible pulpal injury [5]. In contrast, repair has emerged as a minimally invasive, biologically favorable approach that preserves sound restoration and tooth structure [6].

Despite its advantages, the predictability of composite repair remains uncertain due to challenges in achieving durable adhesion to aged composite substrates [7]. Factors such as water sorption and the depletion of unreacted methacrylate groups over time reduce the surface’s chemical reactivity [8]. To counteract these limitations, both mechanical (e.g., air abrasion, diamond bur roughening, laser treatment) and chemical (e.g., acid etching with acidulated phosphate fluoride, hydrofluoric acid, or phosphoric acid) surface treatments have been proposed to enhance micromechanical retention and chemical bonding, particularly when used in conjunction with appropriate adhesive systems [6,9,10].

Among contemporary materials, bulk-fill composites have gained prominence due to their ability to be applied in thicker increments (4–5 mm) without compromising polymerization depth, thereby improving efficiency and reducing technique sensitivity [11]. Their unique formulation—characterized by increased translucency, advanced monomers, and optimized photoinitiators—improves polymerization kinetics and mechanical performance [12]. However, similar to conventional composites, aging alters their surface characteristics, potentially compromising the success of repair.

Although a wide array of surface conditioning techniques and adhesive systems have been advocated to improve bond integrity at the repair interface, consensus on the most effective combination remains elusive. Accordingly, the present study aims to investigate the influence of various surface treatment protocols and adhesive systems on the micro-shear bond strength (μSBS), microtensile bond strength (μTBS), and sealing ability of repaired bulk-fill composites, utilizing micro-computed tomography (micro-CT) for a three-dimensional, non-destructive evaluation of interfacial microleakage. By simultaneously assessing shear bond strength, tensile strength, and microleakage within a unified experimental design, this study offers a novel and comprehensive perspective on adhesive performance in composite repair, yielding original insights with meaningful implications for optimizing clinical protocols.

## 2. Materials and Methods

The materials and adhesive systems used in this study are summarized in Table 1.

### 2.1. Specimen Preparation for Micro-Shear Bond Strength (µSBS)

A total of 72 cylindrical cavities (4 mm diameter × 2 mm depth) were prepared on acrylic resin blocks and restored using a bulk-fill composite (Filtek One Bulk Fill Restorative, 3M ESPE, Seedfeld, Germany), following the manufacturer’s instructions. During placement, a Mylar strip and glass plate were used to ensure a smooth surface and eliminate voids. Specimens were light-cured for 20 s using an LED unit (Woodpecker iLED II; Guilin Woodpecker Medical Instrument Co., Ltd., Guilin, Guangxi, China) and stored in distilled water at 37 °C for 24 h (Nüve Incubator EN 120; NÜVE, Ankara, Turkey). Subsequently, all samples underwent thermocycling (10,000 cycles between 5 °C and 55 °C; dwell time: 30 s; transfer time: 15 s) to simulate intraoral aging (Julabo FT 200; Julabo, Seelbach, Germany).

Following aging, specimens were randomly allocated to three surface treatment groups (*n* = 24):

Group 1 (Control): No surface treatment was applied.

Group 2 (Bur Roughening): Surface roughening was performed using a blue-banded diamond bur in a high-speed handpiece under continuous water and air spray. A new bur was used for every five specimens to maintain consistency.

Group 3 (Air Abrasion): Surface treatment was conducted using 50 µm aluminum oxide (Al_2_O_3_) particles applied at 3 bar for 10 s, with the nozzle held 10 mm from the surface (Prophy-Mate Neo; NSK, Kanuma, Japan).

Each group was subdivided into three adhesive subgroups (*n* = 8) according to the bonding agent applied:

Universal subgroup: A one-step self-etch adhesive (G-Premio Bond; GC Corp., Tokyo, Japan) was applied for 20 s, air-thinned for 5 s, and light-cured for 20 s.

Two-step self-etch subgroup: A two-step self-etch system (Clearfil SE Bond; Kuraray, Osaka, Japan) was used. The primer was applied first, followed by 20 s of gentle air-drying. Then, the bonding agent was applied, air-thinned, and cured for 10 s.

Total-etch subgroup: The composite surface was etched with 37% phosphoric acid (FineEtch 37 Gel; Spident Inc., Incheon, Republic of Korea) for 30 s, rinsed thoroughly, and air-dried. Adper Single Bond 2 (3M ESPE; St. Paul, MN, USA) was then applied with active rubbing for 20 s, gently air-dried for 5 s, and polymerized for 10 s.

**Figure 1:**
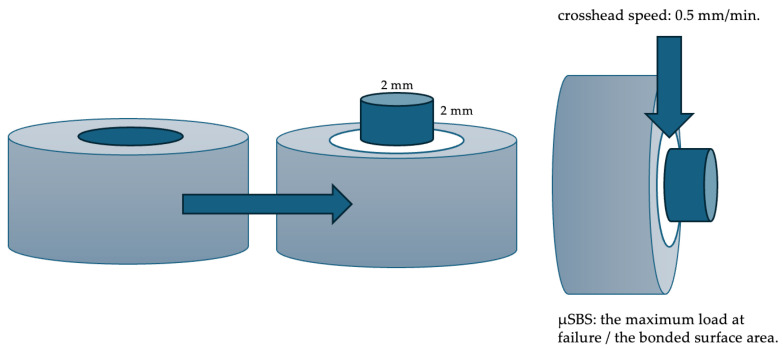
Schematic diagram of shear bond strength specimen preparation and testing procedure.

After surface treatment and adhesive application, a cylindrical increment (2 mm diameter × 2 mm height) of the same composite was placed using a plexiglass mold and light-cured for 20 s (Figure 1). Specimens were then stored in distilled water at 37 °C for 24 h prior to testing.

Shear bond strength was assessed using a universal testing machine (Instron 3345; Instron, Norwood, MA, USA) at a crosshead speed of 0.5 mm/min. The load was applied precisely at the repair interface and the mean slip distance was 5.3 mm. The µSBS values were calculated by dividing the maximum load at failure by the bonded surface area. Failure modes were classified as adhesive, cohesive, or mixed using a stereomicroscope (SZ61; Olympus Co, Tokyo, Japan).

### 2.2. Specimen Preparation for Microtensile Bond Strength (μTBS)

A total of 72 composite beams were fabricated using a bulk-fill composite and a quadrangular mold (6 × 6 × 2 mm). Each mold was half-filled, light-cured for 20 s, and subjected to thermocycling (10,000 cycles; 5–55 °C; 30 s dwell, 15 s transfer) to simulate aging. Aged specimens were randomly assigned to the same surface treatment and adhesive protocols as described in the µSBS section. The molds were then completed with a second composite increment and cured for an additional 20 s. Sixteen composite blocks were sectioned into microtensile beams using a precision cutting machine (Minitom; Struers, Copenhagen, Denmark).

**Figure 2:**
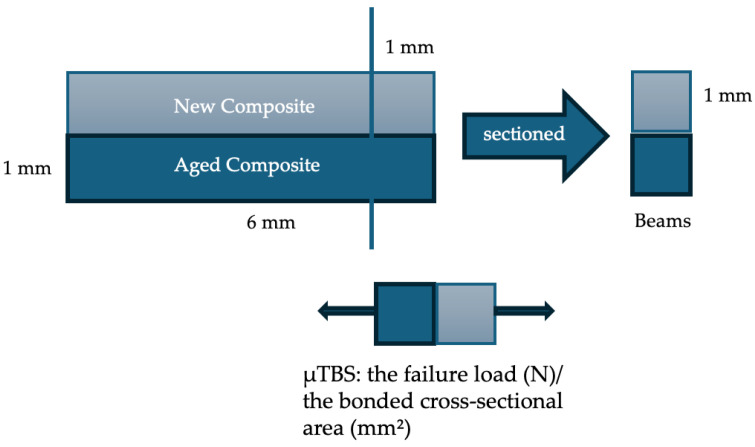
Schematic representation of the microtensile bond strength (µTBS) testing of repaired composite interface.

Microtensile bond strength was measured using a microtensile tester (Bisco Microtensile Tester, Bisco, Schaumburg, IL, USA) at a crosshead speed of 0.5 mm/min (Figure 2). µTBS values were calculated by dividing the failure load (N) by the bonded cross-sectional area (mm^2^), measured precisely with a digital caliper (Powerfix Electronic Digital Caliper, Padget Services, London, UK).

### 2.3. Microleakage Evaluation

Seventy-two composite blocks were fabricated using quadrangular acrylic molds (6 × 6 × 2 mm). Each mold was half-filled with bulk-fill composite, light-cured for 20 s, aged according to the μSBS protocol, and subjected to identical surface treatments and adhesive applications. The molds were then completed with an additional composite increment and cured for another 20 s. After storage in distilled water at 37 °C for 24 h, two layers of nail varnish were applied to all surfaces except a 1 mm margin around the restoration.

The specimens were immersed in 50 wt% ammoniacal silver nitrate solution for 24 h, prepared by dissolving 25 g silver nitrate (Gündüz Chemical, Istanbul, Turkey) in 25 mL distilled water. Following immersion, samples were placed in a developer solution for 8 h, ultrasonically cleaned for 1 min, rinsed, and de-coated with acetone.

Micro-CT analysis was performed using a Bruker Skyscan 1272 system (Coventry, UK) under standardized scanning conditions: an accelerating voltage of 80 kV, beam current of 125 µA, a 0.11 mm Cu filter, and an image resolution of 21.6 µm per pixel. Specimens were scanned through a 360° rotation with angular steps of 0.6°, and an exposure time of 4 s per frame. The average scanning duration per specimen was approximately 1 h, 11 min, and 28 s. The reconstruction of tomographic slices was performed using the manufacturer’s NRecon software Version: 1.7.4.2, yielding approximately 450 cross-sectional images per sample. For evaluation, the slice exhibiting the greatest extent of microleakage was identified and selected. Quantitative assessment of microleakage was conducted based on a predefined scoring scale as follows:

0 = No dye penetration
1 = Dye penetration up to one-half of the repair interface
2 = Dye penetration beyond one-half of the interface, without complete involvement
3 = Complete involvement of the repair interface

For qualitative analysis, the section with maximum leakage was evaluated using ImageJ software (NIH, Bethesda, MD, USA) version 1.53t [13], calibrated with a reference distance and analyzed at 400× magnification.

### 2.4. Statistical Analysis

A priori power analysis was performed using G*Power software (version 3.1, University of Düsseldorf, Düsseldorf, Germany) to determine the minimum required sample size. Based on an effect size of 0.49, a significance level (α) of 0.05, and a statistical power of 80%, the analysis indicated that a minimum of eight specimens per group would be sufficient to detect a statistically significant difference [10]. Data were analyzed using SPSS v23.0 (IBM, Chicago, IL, USA). Normality was assessed via skewness, kurtosis, and the Shapiro–Wilk test. For normally distributed data, ANOVA with Tukey’s post hoc test was used; otherwise, the Kruskal–Wallis and Mann–Whitney U tests were applied. Correlations among the dependent variables—micro-shear bond strength (µSBS), micro tensile bond strength (µTBS), and microleakage—were analyzed in relation to the independent variables, including adhesive strategy and surface treatment method, using correlation coefficients. Results were expressed as mean ± standard deviation, with significance set at *p* < 0.05.

## 3. Results

### 3.1. Micro-Shear Bond Strength (µSBS)

**Figure 3:**
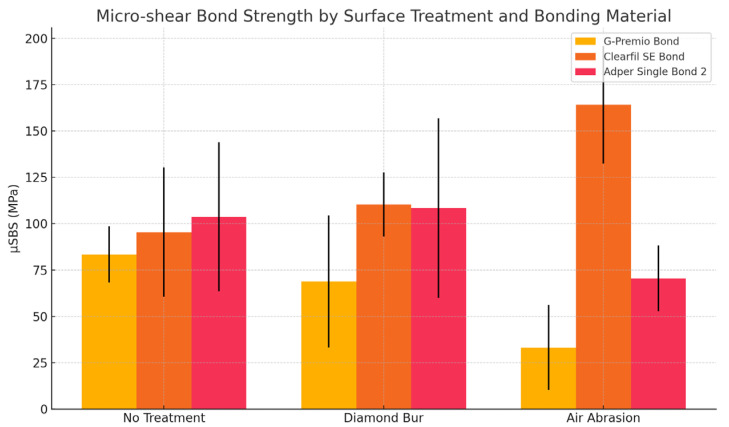
Micro-shear bond strength values (MPa) of different adhesive systems according to surface treatment.

Statistical analysis revealed that the highest µSBS value was observed in the group treated with diamond bur surface conditioning combined with Adper Single Bond 2 (164.1 ± 31.8 MPa), which was significantly higher than that of G-Premio Bond (95.4 ± 34.9 MPa) and Clearfil SE Bond (110.4 ± 17.3 MPa) under the same surface treatment condition (*p* = 0.001), (Table 2). In the absence of surface treatment, Adper Single Bond 2 yielded the lowest µSBS (33.1 ± 22.9 MPa), (Figure 3), significantly lower than both G-Premio Bond (83.4 ± 15.1 MPa) and Clearfil SE Bond (68.9 ± 35.6 MPa), (*p* = 0.002). No statistically significant difference was found between G-Premio Bond and Clearfil SE Bond in the no-treatment group, indicating comparable performance in the absence of surface modification.

**Figure 4:**
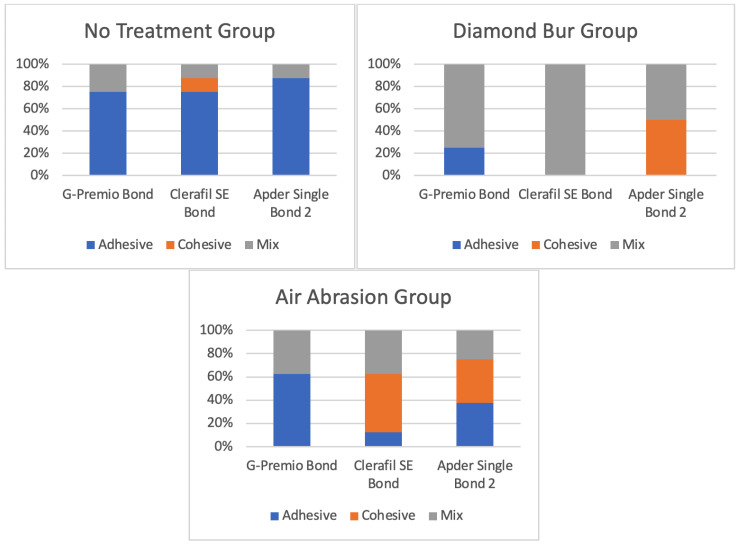
Failure mode of shear bond strength.

**Figure 5:**
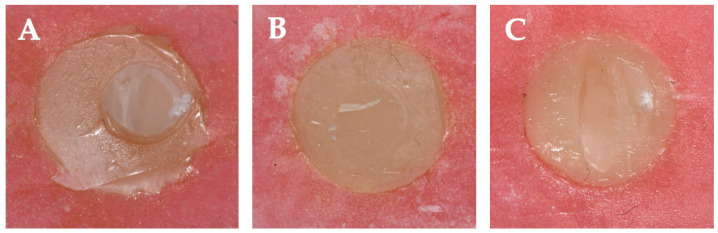
Representative failure modes observed in the micro-shear bond strength test. (**A**) Cohesive failure in the group treated with air abrasion followed by Clearfil SE Bond application; (**B**) adhesive failure in the untreated group with G-Premio Bond application; (**C**) mixed failure in the group with diamond bur surface preparation followed by Adper Single Bond 2 application.

In the air abrasion group, µSBS values ranged from 70.5 ± 17.8 MPa (Adper Single Bond 2) to 108.4 ± 48.5 MPa (Clearfil SE Bond); however, these differences were not statistically significant (*p* = 0.117). Across all bonding agents, surface conditioning with a diamond bur significantly improved µSBS values compared to no treatment (*p* < 0.001), particularly for Adper Single Bond 2 (*p* = 0.001) and Clearfil SE Bond (*p* = 0.046). G-Premio Bond did not show statistically significant differences across the surface treatment protocols (*p* = 0.456). Failure modes were primarily adhesive in the no-treatment group, but with diamond bur roughening, failures shifted towards mixed and cohesive types, especially for Clearfil SE Bond (Figure 4 and Figure 5).

### 3.2. Microtensile Bond Strength (µTBS)

Among all groups, Adper Single Bond 2 combined with diamond bur conditioning yielded the highest µTBS value (35.7 ± 6.7 MPa), significantly surpassing those obtained with G-Premio Bond (15.5 ± 3.9 MPa) and Clearfil SE Bond (22.9 ± 14.7 MPa) under the same surface treatment (*p* < 0.001), (Table 3). Similarly, air abrasion resulted in high bond strengths for Adper Single Bond 2 (33.2 ± 5.1 MPa) and Clearfil SE Bond (30.9 ± 7.3 MPa), both significantly greater than G-Premio Bond (16.6 ± 3.2 MPa) (*p* < 0.001). In the no-treatment group, Adper Single Bond 2 (23.4 ± 6.4 MPa) again demonstrated superior µTBS compared to Clearfil SE Bond (11.7 ± 2.9 MPa) and G-Premio Bond (10.1 ± 1.7 MPa), with the differences reaching statistical significance (*p* < 0.001).

**Figure 6:**
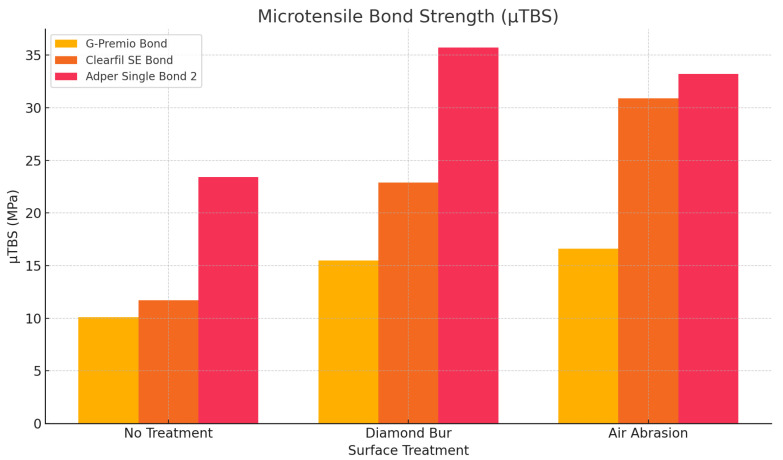
Microtensile bond strength values (MPa) of different adhesive systems according to surface treatment.

When the effect of surface preparation was evaluated within each adhesive, both diamond bur and air abrasion significantly enhanced µTBS compared to no treatment in all adhesive groups (*p* ≤ 0.001). Notably, Clearfil SE Bond showed its highest µTBS with air abrasion (30.9 ± 7.3 MPa), while Adper Single Bond 2 achieved its maximum bond strength following diamond bur treatment (35.7 ± 6.7 MPa), (Figure 6).

### 3.3. Qualitative and Quantitative Microleakage Evaluation

**Figure 7:**
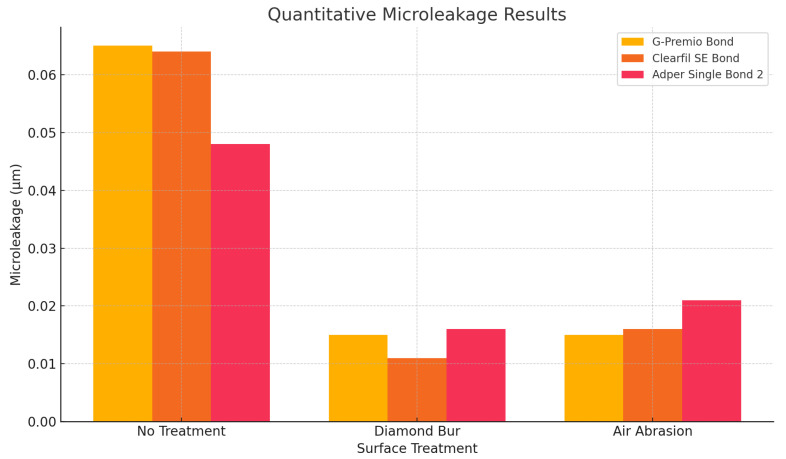
Microleakage results (µm) of different adhesive systems according to surface treatment.

Microleakage values revealed significant differences between adhesives, particularly with surface conditioning (Table 4). The control group showed comparable microleakage values for G-Premio Bond (0.065 ± 0.07 µm) and Clearfil SE Bond (0.064 ± 0.01 µm), while Adper Single Bond 2 showed lower values (0.048 ± 0.05 µm), though differences were not significant (*p* = 0.404). After diamond bur treatment, Clearfil SE Bond showed the lowest microleakage (0.011 ± 0.02 µm), significantly outperforming both G-Premio Bond (0.015 ± 0.01 µm) and Adper Single Bond 2 (0.016 ± 0.02 µm) (*p* < 0.001). In the air abrasion group, microleakage values were similar among the adhesives (*p* = 0.808), (Figure 7).

**Figure 8:**
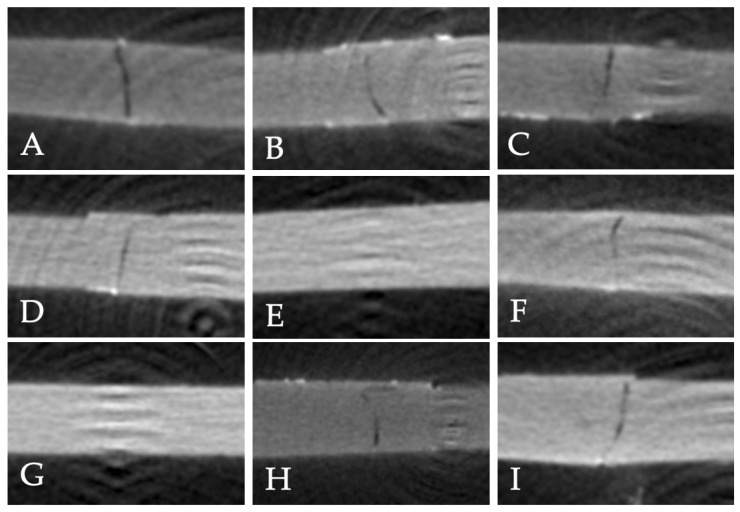
Representative 2D micro-CT images illustrating microleakage patterns in specimens subjected to different surface treatments and adhesive systems. (**A**–**C**): specimens without surface treatment ((**A**): G-Premio Bond; (**B**): Clearfil SE Bond; (**C**): Adper Single Bond 2). (**D**–**F**): specimens subjected to diamond bur roughening ((**D**): G-Premio Bond; (**E**): Clearfil SE Bond; (**F**): Adper Single Bond 2). (**G**–**I**): specimens subjected to air abrasion ((**G**): G-Premio Bond; (**H**): Clearfil SE Bond; (**I**): Adper Single Bond 2).

Qualitative microleakage results showed Adper Single Bond 2 had the highest proportion of specimens with minimal microleakage (score 0) in the control group (62.5%), while G-Premio Bond had a higher proportion of severe leakage (score 3), (Table 5). After diamond bur roughening, both Clearfil SE Bond and Adper Single Bond 2 exhibited minimal microleakage (score 0) in 62.5% of specimens. In the air-abrasion group, Clearfil SE Bond showed the best sealing (75% scoring 1), with Adper Single Bond 2 showing moderate leakage (score 1), (Figure 8).

## 4. Discussion

This study demonstrated that both the surface treatment protocol and the type of adhesive system significantly influenced the µSBS and µTBS, as well as the degree of microleakage at the composite repair interface. These findings highlight the critical role of optimizing surface conditioning and adhesive selection to enhance the longevity and clinical performance of composite repairs.

Composite restorations are susceptible to degradation upon prolonged exposure to the oral environment, wherein various physical and chemical factors may induce alterations in the surface characteristics. Consequently, the aging of restorations must be carefully considered in the planning of repair strategies [14]. A variety of aging methods have been reported in the literature, including water storage, exposure to acidic challenges, thermocycling, and boiling [15]. Previous investigations have consistently indicated that such aging procedures tend to diminish the repair bond strength [14,15]. In accordance with ISO 11405 standards, the use of 500 thermocycles between 5 °C and 55 °C is considered appropriate for simulating short-term aging of dental restorative materials [16]. Additionally, previous studies stated that approximately 10,000 thermocycles would replicate the effects of one year of clinical function, with 20 to 50 cycles equating to the conditions of a single day [17,18,19]. Based on these considerations, the present study employed an extended thermocycling protocol comprising 10,000 cycles to better simulate long-term intraoral aging.

The selection of the composite resin for repair is a critical determinant of the clinical success, with the use of the same composite material as the original restoration generally being recommended [3]. However, previous studies have demonstrated that satisfactory bond strength values can be achieved when bulk-fill composites are repaired with either bulk-fill or conventional composite resins [20,21]. In the present study, to minimize confounding variables associated with material differences, the repair procedures were standardized by utilizing the same bulk-fill composite as that employed in the initial restoration.

Bonding between aged and fresh composite resins relies on macromechanical retention, micromechanical retention, and chemical adhesion [22,23]. Macromechanical retention involves surface roughening (e.g., with a coarse diamond bur), while micromechanical retention is achieved via alumina particle abrasion or acid etching. Furthermore, chemical adhesion is facilitated by primers like silane, which promote bonding between the resin matrix and filler particles [22]. It has been demonstrated that the removal of the superficial, chemically degraded resin layer—formed as a consequence of prolonged intraoral exposure—reveals a cleaner, higher-energy surface. This process enhances surface wettability and increases surface area through the introduction of micro-irregularities, thereby reinforcing the adhesive interface [24]. In this study, surface roughening was performed with blue-coded diamond burs (replaced after every five specimens), and air abrasion was conducted using 50 μm aluminum oxide at 3 bar pressure from 10 mm, following established protocols [22].

Regarding µSBS and µTBS, the untreated groups consistently exhibited the lowest mean values across all adhesive systems tested. This outcome aligns with earlier findings suggesting that aged composite surfaces display reduced surface energy and a diminished number of available unreacted carbon–carbon double bonds, thereby limiting the potential for effective bonding with new composite materials [25]. The inferior bond performance observed in untreated groups is likely attributable to the absence of sufficient micromechanical interlocking and inadequate chemical interaction at the repair interface.

Among the surface pretreatment protocols, both diamond bur and air abrasion significantly improved bond strength values compared to no treatment. These results can be attributed to the ability of these methods to increase surface roughness, thereby enhancing micromechanical interlocking. Specifically, the air-abrasion group in combination with Clearfil SE Bond yielded the highest µSBS (108.4 ± 48.5 MPa), while diamond bur combined with total-etch adhesive provided the highest µSBS (164.1 ± 31.8 N). This suggests that while both surface treatments are beneficial, their efficacy may vary depending on the adhesive system employed. Various clinical studies support the findings of the present investigation [25,26,27]. Similarly, in a study assessing the µSBS of bulk-fill composites repaired using different adhesive protocols, conventional total-etch systems exhibited higher repair bond strengths compared to other adhesive strategies [20].

Çakır et al. reported that universal adhesives exhibited superior bond strength compared to Clearfil SE [28]. Several studies have attributed the improvement in µSBS observed with universal adhesives to the incorporation of 10-MDP, which can establish chemical bonds with various substrates, and silane, which enhances surface wettability [29]. However, the mere presence of 10-MDP cannot be considered a definitive factor, as not all universal adhesives contain 10-MDP at the same concentration or purity. Therefore, it may be inferred that the repair bond strength is influenced by the specific chemical formulation of each adhesive system [30].

In the comparative analyses, total-etch adhesives demonstrated superior performance over both universal and two-step self-etch, particularly with respect to µTBS, likely due to their enhanced demineralization and resin infiltration capabilities. In contrast, Clearfil SE Bond exhibited lower bond strengths in the absence of surface treatment but responded well to mechanical pretreatment, particularly air abrasion. Universal adhesives yielded intermediate outcomes across protocols, potentially reflecting their moderate pH and less aggressive substrate interaction. Nevertheless, in contrast to the findings of the present study, Yin et al. reported that universal adhesives exhibited significantly higher bond strength values compared to the conventional gold-standard adhesive systems, namely the three-step total etch (Scotchbond Muti-purpose) and two-step self-etch (Clearfil SE Bond) protocols [31]. The discrepancy between the results of the current study and those reported by Yin et al. may be attributed to differences in the specific universal composite resins employed for bond strength testing as well as variations in experimental methodologies.

Microleakage assessments revealed that surface conditioning significantly improved the sealing ability of the adhesive systems. Following diamond bur application, Clearfil SE Bond exhibited the lowest microleakage values, outperforming both Adper Single Bond 2 and G-Premio Bond. This finding may be attributed to the chemical composition and functional monomers (e.g., 10-MDP) in Clearfil SE Bond, which enable stronger chemical bonding to the treated composite substrate. In untreated groups, although Adper Single Bond 2 showed numerically lower microleakage values compared to the other adhesives, the differences were not statistically significant, emphasizing the challenges associated with achieving optimal sealing without mechanical preparation. Interestingly, total-etch adhesive Adper Single Bond 2 did not consistently reduce microleakage despite their higher bond strength, indicating that high bond strength does not necessarily translate to superior marginal sealing. Conversely, it should be noted that some studies have reported that variations in adhesive systems do not produce statistically significant differences in microleakage outcomes during composite repair procedures [32,33].

Several limitations of the present study should be acknowledged. First, as an in vitro investigation, the experimental conditions could not fully replicate the complex biological, mechanical, and thermal challenges encountered in the oral environment, such as salivary enzymes, masticatory forces, and biofilm accumulation, which may influence the long-term durability of composite repairs. Additionally, only one type of bulk-fill composite and a limited selection of adhesive systems were evaluated; thus, the findings may not be generalizable to other composite formulations or adhesive materials with different chemical compositions and properties. Furthermore, the use of distilled water for specimen storage, rather than artificial saliva or simulated body fluid, represents another limitation, as it does not reflect the ionic composition, buffering capacity, or pH variations in the oral environment, all of which may affect the degradation behavior and microleakage at the adhesive interface. Future investigations involving different composite types, adhesive systems, and long-term in vivo assessments are warranted to validate and expand upon these findings.

## 5. Conclusions

Within the limitations of this in vitro study, both the surface treatment protocol and adhesive system selection significantly affected composite repair outcomes. Mechanical treatments, particularly diamond bur and air abrasion, enhanced bond strengths compared to no treatment. Adper Single Bond 2 (total etch) yielded the highest bond strengths, especially with diamond bur conditioning. Clearfil SE Bond (self-etch) improved notably after mechanical pretreatment, while G-Premio Bond (universal) showed moderate and variable results. Clearfil SE Bond also demonstrated the best sealing ability following diamond bur application.

Based on these findings, for clinical composite repair procedures, conditioning the aged composite surface with a diamond bur followed by the application of Adper Single Bond 2 (total etch) or Clearfil SE Bond (self-etch) is recommended to optimize bond strength and minimize microleakage.

## Figures and Tables

**Table 1:** The materials and adhesive systems used in the study.

Material	Manifacturer	Composition
Adper Single Bond 2	3M/ESPE, St. Paul, MN, USA	Bis-GMA, HEMA, dimethacrylates, ethanol, water, a novel photoinitiator system, and a methacrylate functional copolymer of polyacrylic and polyitaconic acids, silica nanofiller (5 nm diameter silica particles, 10 wt%).
Clearfill SE Bond	Kuraray, Osaka, Japan	Primer: MDP, HEMA, hydrophilicdimethacrylate, dl-camphorquinone, N,N-diethanol-p-toluidine, water. Bond: MDP, Bis-GMA, HEMA, hydrophobicdimethacrylate, dl-camphorquinone, N,N-diethanol-p-toluidine, silanated colloidalsilica
G-Premio Bond	GC Corporation, Tokyo, Japan	10-MDP, 4-META, 10- methacryoyloxydecyl dihydrogen thiophosphate, methacrylate acid ester, distilled water, acetone, photo-initiators, silica fine powder
Filtek One Bulk Fill Restorative	3M/ESPE, St. Paul, MN, USA	Filler: non-agglomerated/non-aggregated 20 nm silica filler, non-agglomerated/non-aggregated 4 to 11 nm zirconiafiller, aggregated zirconia/silica clusterfiller (comprising 20 nm silica and 4 to 11 nm zirconia particles) and aytterbium trifluoride filler agglomerate 100 nm particles. Matrix: AFM (dynamic stress-relieving monomer), AUDMA, UDMA and 1,12-dodecane-DMA

AFM: addition fragmentation monomers; AUDMA: aromatic urethane dimethacrylate; Bis-GMA: bisphenol-A-glycidyl dimethacrylate; DMA: dimethacrylate; HEMA: hydroxyethyl methacrylate; META: methacryloyloxyethy trimellitate anhydride; 10-MDP: 10-methacryloyloxydecyl dihydrogen phosphate; UDMA: urethane dimethacrylate, wt%: weight percentage.

**Table 2:** Micro-shear bond strength values (MPa).

Repair Protocol	Bonding Materials			*p*
	G-Premio Bond	Clearfil SE Bond	Adper Single Bond 2	
No treatment	83.4 (15.1) ^A^	68.9 (35.6) ^A^	33.1 (22.9) ^a,B^	**0.002** ^†,^*
Diamond bur	95.4 (34.9) ^A^	110.4 (17.3) ^A^	164.1 (31.8) ^b,B^	**0.001** ^‡,^*
Air abrasion	103.7 (40.2)	108.4 (48.5)	70.5 (17.8) ^c^	0.117 ^‡^
***p***	0.456 ^‡^	0.093 ^†^	**0.001** ^‡,^*	

^†^: Kruskal–Wallis, ^‡^: one-way ANOVA. Same lower-case letters indicate no difference between surface preparation protocols; same upper-case letters indicate no difference between bonding materials; * *p* < 0.05.

**Table 3:** Microtensile bond strength values (MPa).

Repair Protocol	Bonding Materials			*p*
	G-Premio Bond	Clearfil SE Bond	Adper Single Bond 2	
No treatment	10.1 (1.7) ^a,A^	11.7 (2.9) ^a,A^	23.4 ± 6.4 ^a,B^	**<0.001** *
Diamond bur	15.5 (3.9) ^b,A^	22.9 (14.7) ^b,A^	35.7 ± 6.7 ^b,B^	**<0.001** *
Air abrasion	16.6 (3.2) ^b,A^	30.9 (7.3) ^c,B^	33.2 ± 5.1 ^b,B^	**<0.001** *
*p*	**0.001** *	**<0.001** *	**0.001** *	

One-way ANOVA. Same lower-case letters indicate no difference between surface preparation protocols; same upper-case letters indicate no difference between bonding materials. * *p* < 0.05.

**Table 4:** Microleakage values in µm.

Repair Protocol	Bonding Materials			*p*
	G-Premio Bond	Clearfil SE Bond	Adper Single Bond 2	
No treatment	0.065 (0.07)	0.064 (0.01) ^a^	0.048 (0.05)	0.404
Diamond bur	0.015 (0.01)	0.011 (0.02) ^b^	0.016 (0.02)	0.626
Air abrasion	0.015 (0.02)	0.016 (0.02) ^b^	0.021 (0.01)	0.808
***p***	0.269	**<0.001** *	0.389	

Kruskall–Wallis test. Same lower-case letters indicate no difference between surface preparation protocols. * *p* < 0.05.

**Table 5:** Distribution of microleakage scores.

Repair Protocol	Bonding Materials	Scores			
		0	1	2	3
No treatment	G-Premio Bond	3 (37.5%)	-	3 (37.5%)	2 (25%)
	Clearfil SE Bond	-	6 (75%)	2 (25%)	-
	Adper Single Bond 2	5 (62.5%)	1 (12.5%)	2 (25%)	-
Diamond bur	G-Premio Bond	3 (37.5%)	5 (62.5%)	-	-
	Clearfil SE Bond	5 (62.5%)	3 (37.5%)	-	-
	Adper Single Bond 2	5 (62.5%)	3 (37.5%)		-
Air abrasion	G-Premio Bond	5 (62.5%)	3 (37.5%)	-	-
	Clearfil SE Bond	6 (75%)	2 (25%)	-	-
	Adper Single Bond 2	3 (37.5%)	5 (62.5%)	-	-

## Data Availability

The data presented in this study are available on request from the corresponding author.

## Abbreviations

The following abbreviations are used in this manuscript:

µSBS	Micro-shear bond strength
µTBS	Microtensile bond strength
10-MDP	10-methacryloyloxydecyl dihydrogen phosphate

## References

[1] Hickel R., Brüshaver K., Ilie N. (2013). Repair of restorations—criteria for decision making and clinical recommendations. Dent. Mater..

[2] Gordan V.V., Mjör I.A., Blum I.R., Wilson N. (2003). Teaching students the repair of resin-based composite restorations: A survey of North American dental schools. J. Am. Dent. Assoc..

[3] Blum I.R., Lynch C.D., Wilson N.H. (2014). Factors influencing repair of dental restorations with resin composite. Clin. Cosmet. Investig. Dent..

[4] Al-Asmar A.A., Al-Hiyasat A.S., Pitts N.B. (2022). Reframing perceptions in operative dentistry relating evidence-based dentistry and clinical decision making: A cross-sectional study among Jordanian dentists. BMC Oral Health.

[5] Blum I.R., Özcan M. (2018). Reparative Dentistry: Possibilities and Limitations. Curr. Oral Health Rep..

[6] Ömeroğlu M.K., Çam M., Doğruer I., Kaynar Z.B. (2025). The effect of different surface treatments and adhesive systems on shear bond strength in universal nanohybrid composite resin repair. BMC Oral Health.

[7] Neto H.N.M., Leite J.V.C., de Medeiros J.M., Muniz I.D., De Andrade A.K., Duarte R.M., De Souza G.M., Lima R.B. (2024). Scoping review: Effect of surface treatments on bond strength of resin composite repair. J. Dent..

[8] Altinci P., Mutluay M., Tezvergil-Mutluay A. (2018). Repair bond strength of nanohybrid composite resins with a universal adhesive. Acta Biomater. Odontol. Scand..

[9] Özcan M., Corazza P.H., Marocho S.M., Barbosa S.H., Bottino M.A. (2013). Repair bond strength of microhybrid, nanohybrid and nanofilled resin composites: Effect of substrate resin type, surface conditioning and ageing. Clin. Oral Investig..

[10] de Jesus Tavarez R.R., Almeida Júnior L., Guará T.C.G., Ribeiro I.S., Maia Filho E.M., Firoozmand L.M. (2017). Shear bond strength of different surface treatments in bulk fill, microhybrid, and nanoparticle repair resins. Clin. Cosmet. Investig. Dent..

[11] Ahlholm P., Staxrud F., Sipilä K., Vallittu P. (2023). Repair bond strength of bulk-fill composites: Influence of different primers and direction of debonding stress. Biomater. Investig. Dent..

[12] Aquino C., Mathias C., Barreto S.C., Cavalcanti A.N., Marchi G.M., Mathias P. (2020). Repair Bond Strength and Leakage of Non-Aged and Aged Bulk-fill Composite. Oral Health Prev. Dent..

[13] Schneider C.A., Rasband W.S., Eliceiri K.W. (2012). NIH Image to ImageJ: 25 years of image analysis. Nat. Methods.

[14] Celik E.U., Ergücü Z., Türkün L.S., Ercan U.K. (2011). Tensile bond strength of an aged resin composite repaired with different protocols. J. Adhes. Dent..

[15] Özcan M., Barbosa S.H., Melo R.M., Galhano G.A., Bottino M.A. (2007). Effect of surface conditioning methods on the microtensile bond strength of resin composite to composite after aging conditions. Dent. Mater..

[16] Hadilou M., Dolatabadi A., Ghojazadeh M., Hosseinifard H., Alizadeh Oskuee P., Pournaghi Azar F. (2022). Effect of Different Surface Treatments on the Long-Term Repair Bond Strength of Aged Methacrylate-Based Resin Composite Restorations: A Systematic Review and Network Meta-analysis. Biomed. Res. Int..

[17] Ghavami-Lahiji M., Firouzmanesh M., Bagheri H., Jafarzadeh Kashi T.S., Razazpour F., Behroozibakhsh M. (2018). The effect of thermocycling on the degree of conversion and mechanical properties of a microhybrid dental resin composite. Restor. Dent. Endod..

[18] Gale M.S., Darvell B.W. (1999). Thermal cycling procedures for laboratory testing of dental restorations. J. Dent..

[19] Andrade de Freitas S.L., Brandt W.C., Miranda M.E., Vitti R.P. (2018). Effect of Thermocycling, Teeth, and Polymerization Methods on Bond Strength Teeth-Denture Base. Int. J. Dent..

[20] Akgül S., Kedici Alp C., Bala O. (2021). Repair potential of a bulk-fill resin composite: Effect of different surface-treatment protocols. Eur. J. Oral Sci..

[21] Cuevas-Suárez C.E., Nakanishi L., Isolan C.P., Ribeiro J.S., Moreira A.G., Piva E. (2020). Repair bond strength of bulk-fill resin composite: Effect of different adhesive protocols. Dent. Mater. J..

[22] Loomans B., Özcan M. (2016). Intraoral Repair of Direct and Indirect Restorations: Procedures and Guidelines. Oper. Dent..

[23] Cavalcanti A.N., De Lima A.F., Peris A.R., Mitsui F.H., Marchi G.M. (2007). Effect of surface treatments and bonding agents on the bond strength of repaired composites. J. Esthet. Restor. Dent..

[24] Costa T.R., Ferreira S.Q., Klein-Júnior C.A., Loguercio A.D., Reis A. (2010). Durability of surface treatments and intermediate agents used for repair of a polished composite. Oper. Dent..

[25] Wendler M., Belli R., Panzer R., Skibbe D., Petschelt A., Lohbauer U. (2016). Repair Bond Strength of Aged Resin Composite after Different Surface and Bonding Treatments. Materials.

[26] Martin J., Fernandez E., Estay J., Gordan V.V., Mjör I.A., Moncada G. (2013). Minimal invasive treatment for defective restorations: Five-year results using sealants. Oper. Dent..

[27] Baur V., Ilie N. (2013). Repair of dental resin-based composites. Clin. Oral Investig..

[28] Çakir N.N., Demirbuga S., Balkaya H., Karadaş M. (2018). Bonding performance of universal adhesives on composite repairs, with or without silane application. J. Conserv. Dent..

[29] Nagaoka N., Yoshihara K., Feitosa V.P., Tamada Y., Irie M., Yoshida Y., Van Meerbeek B., Hayakawa S. (2017). Chemical interaction mechanism of 10-MDP with zirconia. Sci. Rep..

[30] Carrilho E., Cardoso M., Marques Ferreira M., Marto C.M., Paula A., Coelho A.S. (2019). 10-MDP Based Dental Adhesives: Adhesive Interface Characterization and Adhesive Stability—A Systematic Review. Materials.

[31] Yin H., Kwon S., Chung S.H., Kim R.J.Y. (2022). Performance of Universal Adhesives in Composite Resin Repair. Biomed. Res. Int..

[32] Cavalcanti A.N., Lavigne C., Fontes C.M., Mathias P. (2004). Microleakage at the composite-repair interface: Effect of different adhesive systems. J. Appl. Oral Sci..

[33] de Medeiros T.C., de Lima M.R., Bessa S.C., de Araújo D.F., Galvão M.R. (2019). Repair bond strength of bulk fill composites after different adhesion protocols. J. Clin. Exp. Dent..

